# Rapid Microwave-Assisted Synthesis of ZnIn_2_S_4_ Nanosheets for Highly Efficient Photocatalytic Hydrogen Production

**DOI:** 10.3390/nano13131957

**Published:** 2023-06-27

**Authors:** Yu-Cheng Chang, Yung-Chang Chiao, Po-Chun Hsu

**Affiliations:** 1 Department of Materials Science and Engineering, Feng Chia University, Taichung 407102, Taiwan; d0455077@gmail.com; 2 Pritzker School of Molecular Engineering, University of Chicago, Chicago, IL 60637, USA; pochunhsu@uchicago.edu

**Keywords:** ZnIn_2_S_4_ nanosheets, microwave-assisted synthesis, sacrificial reagents, photocatalytic hydrogen production, seawater

## Abstract

In this study, a facile and rapid microwave-assisted synthesis method was used to synthesize In_2_S_3_ nanosheets, ZnS nanosheets, and ZnIn_2_S_4_ nanosheets with sulfur vacancies. The two-dimensional semiconductor photocatalysts of ZnIn_2_S_4_ nanosheets were characterized by XRD, FESEM, BET, TEM, XPS, UV–vis diffuse reflectance, and PL spectroscopy. The ZnIn_2_S_4_ with sulfur vacancies exhibited an evident energy bandgap value of 2.82 eV, as determined by UV–visible diffuse reflectance spectroscopy, and its energy band diagram was obtained through the combination of XPS and energy bandgap values. ZnIn_2_S_4_ nanosheets exhibited about 33.3 and 16.6 times higher photocatalytic hydrogen production than In_2_S_3_ nanosheets and ZnS nanosheets, respectively, under visible-light irradiation. Various factors, including materials, sacrificial reagents, and pH values, were used to evaluate the influence of ZnIn_2_S_4_ nanosheets on photocatalytic hydrogen production. In addition, the ZnIn_2_S_4_ nanosheets revealed the highest photocatalytic hydrogen production from seawater, which was about 209.4 and 106.7 times higher than that of In_2_S_3_ nanosheets and ZnS nanosheets, respectively. The presence of sulfur vacancies in ZnIn_2_S_4_ nanosheets offers promising opportunities for developing highly efficient and stable photocatalysts for photocatalytic hydrogen production from seawater under visible-light irradiation.

## 1. Introduction

Microwave-assisted synthesis is a popular technique for the rapid and facile preparation of organic/inorganic nanostructured materials in various media [1,2]. In addition, it offers control for internal and volumetric heating of materials, making it an environmentally friendly method for fabricating new material structures, components, and devices [3]. Microwave-assisted synthesis has been widely used to synthesize nanostructured materials, including metals, nanoporous materials, colloidal nanocrystals, polymer nanocomposites, and inorganic or semiconducting nanomaterials [2,4,5,6,7,8,9]. Microwave-assisted synthesis of nanostructures offers several advantages, including contactless heat transfer to the reactant sample, short reaction time, high selectivity and yield, energy efficiency, uniform and selective distribution of energy, control over size and temperature, improved safety and reproducibility, and excellent control over experimental parameters, making it an inexpensive, quick, clean, and versatile technique for the preparation of nanostructures [10,11,12,13,14]. In addition, microwave-assisted synthesis has distinct characteristics that render it viable for achieving extensive-scale industrial manufacturing [15,16].

Zinc indium sulfide (ZnIn_2_S_4_) is a highly efficient two-dimensional semiconductor photocatalyst from the AB_2_X_4_ family of ternary compounds, widely used in solar energy conversion and environmental purification [17,18,19,20]. ZnIn_2_S_4_ typically forms two-dimensional nanosheets due to its layered crystal structure, but these nanosheets quickly aggregate and assemble into three-dimensional nanostructures to minimize the surface energy for most reported ZnIn_2_S_4_ [19,21]. ZnIn_2_S_4_ has two crystal structures: hexagonal and cubic, with hexagonal ZnIn_2_S_4_ being the most researched in photocatalysis [22,23,24]. Recently, ZnIn_2_S_4_ has gained attention in photocatalytic hydrogen production, carbon dioxide conversion, and pollutant removal, owing to its excellent light absorption and strong redox capabilities [25,26,27,28]. However, the low separation degree of photogenerated charge carriers and the low electron-transport efficiency of ZnIn_2_S_4_ limit its application in photocatalysis [29]. In order to improve photocatalytic efficiency, introducing sulfur or zinc vacancies has proven effective [30,31]. So far, most of the research on ZnIn_2_S_4_ has focused on combining different materials to improve its photocatalytic hydrogen production performance, but there are few reports on preparing ZnIn_2_S_4_ with sulfur defects via the microwave-assisted synthesis method [32].

Previous research has reported significant instances of photocatalytic hydrogen production, predominantly employing deionized or pure water [33,34]. Nevertheless, the preparation of deionized or pure water necessitates the purification of fresh water, which results in energy wastage and contributes to the depletion of freshwater resources [35,36]. Furthermore, the increasing occurrence of abnormal climate changes, such as heavy rainfall or drought, has led to a scarcity of freshwater resources [37,38]. Consequently, developing a photocatalyst capable of effectively decomposing seawater to generate hydrogen would address this challenge and further enhance the situation [39]. Therefore, this study aimed to produce hydrogen through the photocatalytic decomposition of seawater using ZnIn_2_S_4_ nanosheets with sulfur defects, potentially reducing freshwater consumption and addressing the scarcity of freshwater resources.

This study successfully synthesized In_2_S_3_ nanosheets, ZnS nanosheets, and ZnIn_2_S_4_ nanosheets with sulfur vacancies for photocatalytic hydrogen production by microwave-assisted synthesis. The photocatalytic activities of ZnIn_2_S_4_ nanosheets with sulfur vacancies were approximately 33.3 times higher than those of In_2_S_3_ nanosheets and about 16.6 times higher than those of ZnS nanosheets under visible-light irradiation. Notably, utilizing ZnIn_2_S_4_ nanosheets with sulfur vacancies as a photocatalyst demonstrated their exceptional performance in efficiently splitting seawater under visible-light irradiation.

## 2. Materials and Methods

### 2.1. Chemicals

Anhydrous zinc chloride (ZnCl_2_, 98%), anhydrous indium(III) chloride (InCl_3_, 98%), thioacetamide (TAA, C_2_H_5_NS, 98%), sodium sulfide nonahydrate (Na_2_S·9H_2_O, 98%), sodium sulfite (Na_2_SO_3_, 98%), methanoic acid (CH_2_O_2_, 97%), folic acid dihydrate (C_19_H_19_N_7_O_6_∙2H_2_O, 97%), methanol (CH_3_OH, 99%), and sodium chloride (NaCl, 99%) were purchased from Alfa Aesar (Haverhill, MA, USA) without further purification. Deionized water (>18 MΩ·cm) was used throughout the experimental processes.

### 2.2. Synthesis of In_2_S_3_, ZnIn_2_S_4_, and ZnS Nanosheets

A facile microwave-assisted synthesis method can be used to synthesize In_2_S_3_ nanosheets, ZnIn_2_S_4_ nanosheets, and ZnS nanosheets. For In_2_S_3_ nanosheets, 0.0045 g of InCl_3_ and 0.0075 g of TAA were dissolved in the 20 mL solvent (deionized water: ethanol = 3:1) and put into a Teflon-lined digestion vessel. For ZnIn_2_S_4_ nanosheets, 0.0068 g of ZnCl_2_, 0.0045 g of InCl_3_, and 0.0075 gTAA were dissolved in the 20 mL solvent (deionized water: ethanol = 3:1) and put into the Teflon-lined digestion vessel. For ZnS nanosheets, 0.0136 g of ZnCl_2,_ and 0.0075 g TAA were dissolved in the 20 mL solvent (deionized water: ethanol = 3:1) and put into the Teflon-lined digestion vessel. A safety shield was placed around the vessel after sealing the reaction solution inside the vessel, using the vessel cover as an overpressure release valve. The vessel was then heated at 140 °C for 1 h using a microwave synthesizer (ETHOS EASY, Milestone, Sorisole, Italy). Following the cooling stage, the as-prepared samples were thoroughly cleaned with deionized water, purified through centrifugation, and dried at 70 °C for 2 h.

### 2.3. Characterization

The crystal structure of the as-prepared photocatalysts was investigated by X-ray diffractometry (XRD) using a Bruker D2 phaser system (Billerica, MA, USA) in the 2θ range of 25–65° with Cu Kα (λ = 1.5418 Å). In addition, field-emission scanning electron microscopy (FESEM) was used to investigate the surface morphology of the as-prepared photocatalysts using a Hitachi S-4800 microscope (Tokyo, Japan) operating at a 15 kV accelerating voltage. The specific surface areas of the as-prepared photocatalysts were measured using the nitrogen adsorption technique (ASAP 2020, Micromeritics, GA, USA). Field-emission transmission electron microscopy (FETEM) at an accelerating voltage of 200 kV was employed to characterize the microstructures and composition of the ZnIn_2_S_4_ nanosheets using a JEOL 2100F microscope (Tokyo, Japan). The surface chemical composition and sulfur vacancies of the ZnIn_2_S_4_ nanosheets were detected by using X-ray photoelectron spectroscopy (XPS, ULVAC-PHI PHI 5000 Versaprobe II system, Chigasaki, Japan) with an Al K source. The as-prepared photocatalysts’ UV–vis diffuse reflectance spectra were recorded using a UV–vis spectrophotometer (PerkinElmer Lambda 650 S, Waltham, MA, USA) and analyzed using a Protrustech MRI532S instrument (Tainan, Taiwan). A three-electrode system was utilized with the assistance of the Zennium electrochemical workstation (Zahhner, Kronach, Germany) for measuring electrochemical impedance spectroscopy (EIS).

### 2.4. Photocatalytic Hydrogen Production Experiment

In order to investigate photocatalytic hydrogen production, an experiment was conducted with the PCX50B Discover photocatalytic reaction system (Perfect Light Technology, Beijing, China). For the experiment, 25 mg of the as-prepared photocatalysts was mixed with 50 mL of different water bases, including deionized water, reverse-osmosis water, tap water, and seawater. Different sacrificial agents, such as sodium sulfide, sodium sulfate, methanol, and methanoic acid, were added to the water bases at 0.1 M concentrations. The mixtures were then placed in 60 mL quartz tubes, and Ar gas was introduced for 30 min to eliminate air. The tubes were sealed with rubber stoppers and irradiated for 3 h using a 5 W blue LED (λ = 420 nm) as a visible-light source. The hydrogen produced was quantified using a gas chromatograph equipped with a thermal conductivity detector (TCD).

## 3. Results and Discussion

**Figure 1:**
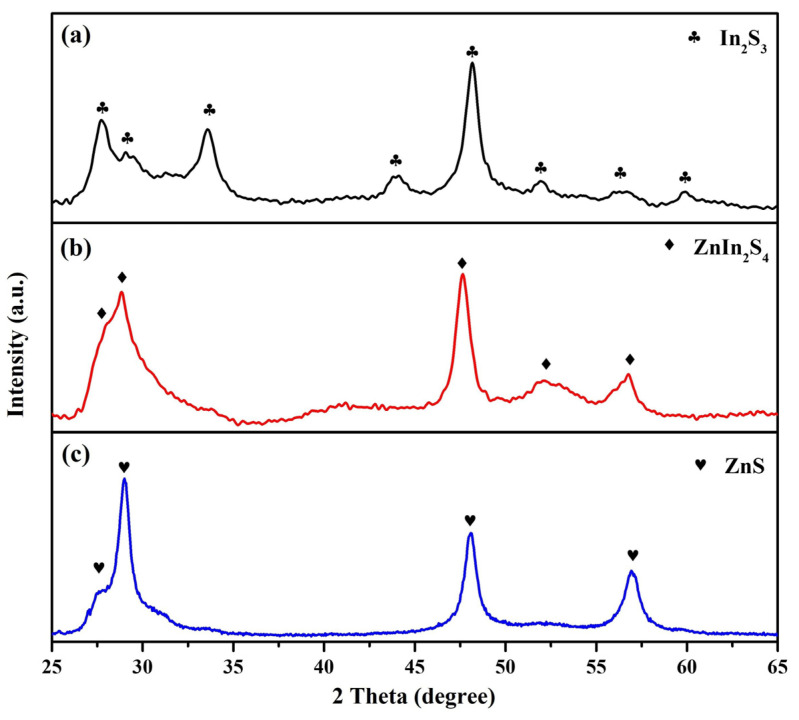
The XRD patterns of (**a**) In_2_S_3_ nanosheets, (**b**) ZnIn_2_S_4_ nanosheets, and (**c**) ZnS nanosheets.

The crystalline phases of the prepared photocatalysts were analyzed using X-ray diffraction (XRD). Figure 1 shows the XRD patterns of In_2_S_3_ nanosheets, ZnIn_2_S_4_ nanosheets, and ZnS nanosheets grown via rapid microwave-assisted synthesis at 140 °C for 1 h. For In_2_S_3_ nanosheets (Figure 1a), the eight peaks at 27.7°, 28.9°, 33.6°, 43.9°, 48.0°, 51.9°, 56.3°, and 59.8° correspond to the (311), (222), (400), (511), (440), (610), (533), and (444) planes of typical cubic In_2_S_3_ (JCPDS card No. 32-0456), respectively. For ZnIn_2_S_4_ nanosheets (Figure 1b), the five peaks at 27.7°, 28.8°, 47.3°, 52.2°, and 56.4°correspond to the (102), (103), (110), (1012), and (203) planes of typical hexagonal ZnIn_2_S_4_ (JCPDS card No. 72-0773), respectively. For ZnS nanosheets (Figure 1c), the four peaks at 27.7°, 28.8°, 47.3°, and 56.4°correspond to the (100), (002), (110), and (112) planes of typical hexagonal ZnS (JCPDS card No. 80-0007), respectively. The absence of diffraction peaks corresponding to impurities further confirms the phase purity of the prepared photocatalysts.

**Figure 2:**
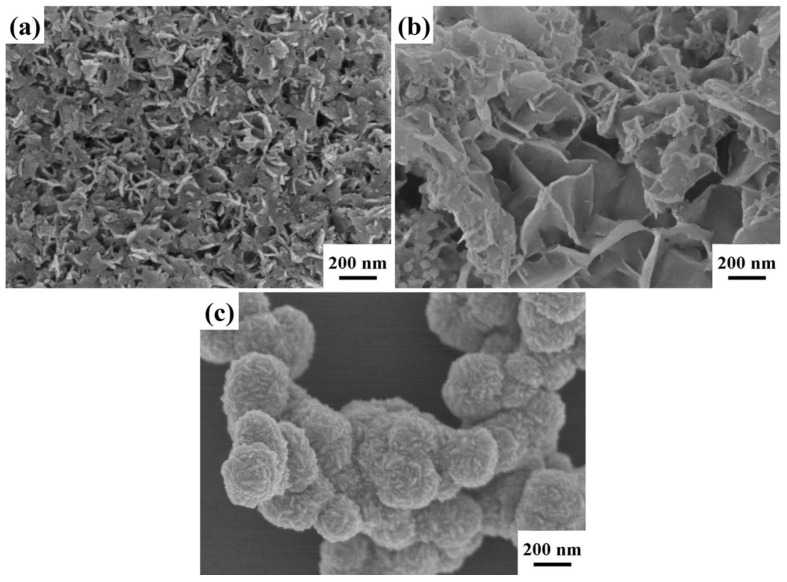
The FESEM images of (**a**) In_2_S_3_ nanosheets, (**b**) ZnIn_2_S_4_ nanosheets, and (**c**) ZnS nanosheets.

The surface morphologies of the synthesized In_2_S_3_ nanosheets, ZnIn_2_S_4_ nanosheets, and ZnS nanosheets were characterized using FESEM, as depicted in Figure 2. In Figure 2a, small and interconnected In_2_S_3_ nanosheets can be observed, and the thickness of the nanosheets is about 15–40 nm. ZnIn_2_S_4_ nanosheets are larger and interconnected, with a thickness of about 15–60 nm, as shown in Figure 2b. Figure 2c reveals that the ZnS nanosheets are stacked on one another and form a spherical structure. We utilized a BET (Brunauer–Emmett–Teller) analyzer to examine the specific surface areas of In_2_S_3_ nanosheets, ZnIn_2_S_4_ nanosheets, and ZnS nanosheets. The nitrogen adsorption–desorption isotherms revealed that the specific surface areas of these photocatalysts were as follows: 99.8 (In_2_S_3_ nanosheets), 77.3 (ZnIn_2_S_4_ nanosheets), and 35.8 (ZnS nanosheets) m^2^g^−1^. The specific surface areas of the In_2_S_3_ nanosheets and ZnIn_2_S_4_ nanosheets were significantly larger than that of the ZnS nanosheets, which is consistent with the FESEM characterization results.

**Figure 3:**
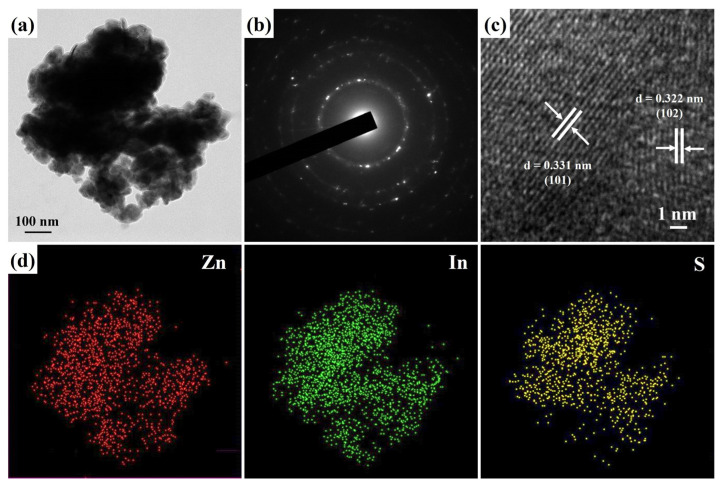
(**a**) FETEM, (**b**) SAED, (**c**) HRTEM, and (**d**) EDS mapping images of ZnIn_2_S_4_ nanosheets.

Figure 3a shows the FETEM image of ZnIn_2_S_4_ nanosheets, which is consistent with the FESEM image of the nanosheet structure made of lamellar stacks. Additionally, the polycrystalline nature of the ZnIn_2_S_4_ nanosheets is substantiated by the presence of characteristic polycrystalline diffraction rings observed in the selected-area electron diffraction (SAED) patterns, as shown in Figure 3b. The prominent diffraction ring patterns correspond to the crystal planes (102), (103), (110), (1012), and (203), which are indicative of the typical hexagonal structure of ZnIn_2_S_4_ (JCPDS card No. 72-0773). These findings are consistent with the results obtained from the XRD analysis, confirming the polycrystalline properties of the ZnIn_2_S_4_ nanosheets. Figure 3c reveals that the HRTEM image of the lattice fringes of 0.331 nm and 0.322 nm are assigned to the (101) and (102) planes of hexagonal ZnIn_2_S_4_, respectively. Moreover, the mapping images obtained from the energy-dispersive spectrometry (EDS) analysis (Figure 3d) reveal a uniform and interconnected distribution of elemental Zn, In, and S. These results provide conclusive evidence of the successful synthesis of ZnIn_2_S_4_ nanosheets.

**Figure 4:**
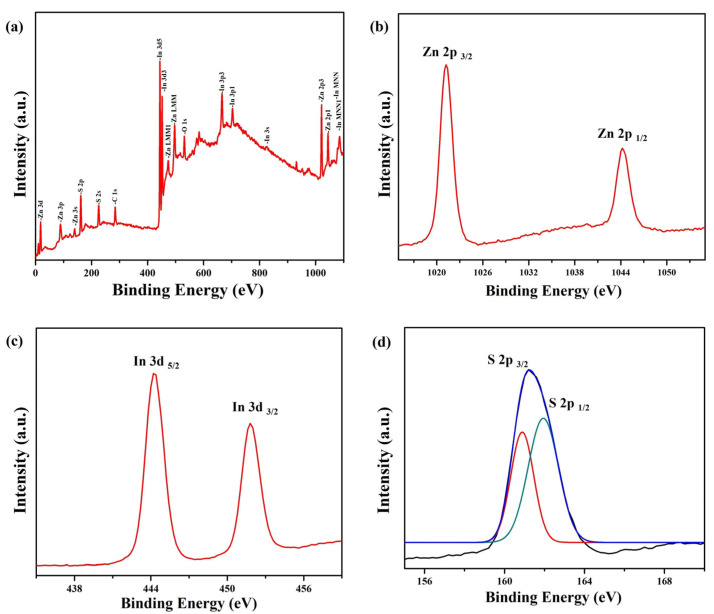
XPS spectra of the ZnIn_2_S_4_ nanosheets: (**a**) survey spectrum, (**b**) Zn 2p, (**c**) In 3d, and (**d**) S 2p.

X-ray photoelectron spectroscopy (XPS) was utilized to determine the elemental composition and chemical configurations of the ZnIn_2_S_4_ nanosheets. Figure 4a displays the complete XPS spectrum, which confirms the presence of elemental Zn, In, S, C, and O in the ZnIn_2_S_4_ nanosheets. The appearance of C 1s and O 1s signals can be explained by the presence of pump oil in the XPS equipment’s vacuum system and the adsorption of oxygen atoms, respectively. Figure 4b shows that the high-resolution XPS spectrum of Zn 2p reveals two peaks at 1021.2 eV and 1044.2 eV, which are assigned to Zn 2p_3/2_ and Zn 2p_1/2_, respectively, indicating the presence of Zn^2+^ in ZnIn_2_S_4_. Figure 4c shows that the high-resolution XPS spectrum of In 3d reveals two peaks at 444.2 eV and 451.8 eV, which are assigned to In 3d_5/2_ and In 3d_3/2_, respectively, indicating the presence of In^3+^ in ZnIn_2_S_4_. Compared with pure ZnIn_2_S_4_ (S 2p_1/2_ = 161.4 eV and S 2p_3/2_ = 162.7 eV), the XPS peaks of S 2p_1/2_ and 2p_3/2_ in ZnIn_2_S_4_ nanosheets with sulfur defects shift to lower binding energies of 160.9 and 161.9 eV, respectively [25,31,40]. This peak shift can primarily be attributed to the presence of sulfur vacancies, indicating the influence of these vacancies on the electronic structure of the materials [41]. This result demonstrates that rapid microwave-assisted synthesis can facilitate the formation of ZnIn_2_S_4_ nanosheets with sulfur defects during the same reaction process.

**Figure 5:**
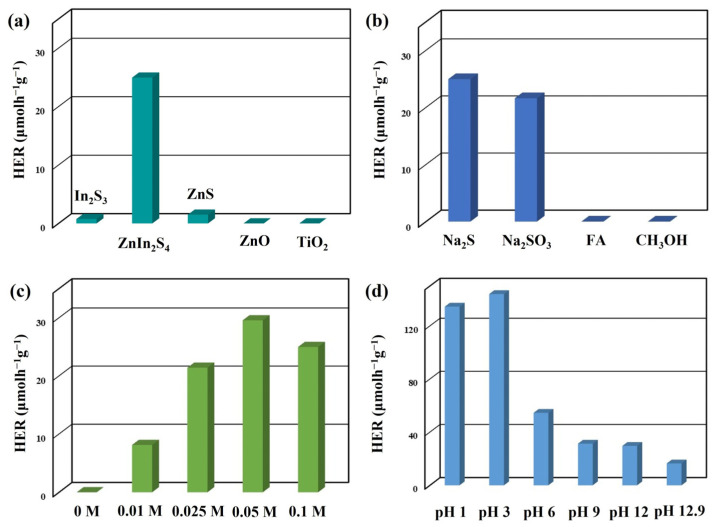
(**a**) The average HER of different photocatalysts. The average HER of ZnIn_2_S_4_ nanosheets under the different photocatalytic reaction conditions: (**b**) sacrificial reagents, (**c**) Na_2_S concentrations, and (**d**) pH values.

In order to assess the photocatalytic characteristics of the catalyst, a photocatalytic hydrogen evolution experiment was conducted in deionized water. The hydrogen evolution rate (HER) activities of the as-prepared photocatalysts (In_2_S_3_ nanosheets, ZnIn_2_S_4_ nanosheets, and ZnS nanosheets) and commercial photocatalysts (ZnO nanopowder and TiO_2_ nanopowder) were evaluated under visible-light irradiation (5 W blue LED, λ = 420 nm) using 0.1 M Na_2_S as a sacrificial reagent in deionized water with pH = 12, as shown in Figure 5a. The ZnIn_2_S_4_ nanosheets exhibited an HER value of 24.95 μmolh^−1^g^−1^, which is about 33.3 and 16.6 times higher than 0.75 (In_2_S_3_ nanosheets) and 1.5 (ZnS nanosheets) μmolh^−1^g^−1^, respectively. On the other hand, the pure ZnIn_2_S_4_ nanosheets synthesized by a hydrothermal method revealed significantly lower HER values of 0.065 μmolh^−1^g^−1^ under the same photocatalytic reaction conditions [25]. These outcomes indicate that utilizing the rapid microwave-assisted synthesis approach facilitates the growth of ZnIn_2_S_4_ nanosheets with sulfur vacancies, leading to a notable improvement in their photocatalytic hydrogen evolution.

In photocatalytic hydrogen production, sacrificial reagents are often employed to enhance the efficiency of oxidation reactions in aqueous environments, compensating for the inefficiency of pure water oxidation [42]. Figure 5b illustrates the HER values of ZnIn_2_S_4_ nanosheets under visible-light irradiation using four different sacrificial reagents: sodium sulfide (Na_2_S), sodium sulfite (Na_2_SO_3_), folic acid (FA), and methanol (CH_3_OH). The HER values of the ZnIn_2_S_4_ nanosheets were 24.95 (Na_2_S), 21.6 (Na_2_SO_3_), 0 (FA), and 0 μmolh^−1^g^−1^ (CH_3_OH). In photocatalytic hydrogen generation, the sacrificial agent serves the dual role of providing electrons for proton reduction and scavenging holes to impede the recombination of electron–hole pairs, thereby improving the process’s efficiency [43,44]. Consequently, using sodium sulfide or sodium sulfate as a sacrificial reagent can enhance the reduction/oxidation reaction, reduce the photocorrosion of metal sulfide materials, and enhance their photocatalytic hydrogen production capability [42,45].

In general, concentrated sacrificial reagents are preferable, as they promote better diffusion of the reacting species toward the surface of the photocatalysts [46,47]. However, it is essential to note that the highest hydrogen evolution rate cannot be achieved with diluted or highly concentrated sacrificial reagents [48]. The impact of Na_2_S concentrations on the photocatalytic efficiency of ZnIn_2_S_4_ nanosheets is shown in Figure 5c. The average HER values of the ZnIn_2_S_4_ nanosheets were recorded as follows: 0 (without Na_2_S), 8.1 (0.01 M Na_2_S), 21.4 (0.025 M Na_2_S), 29.55 (0.05 M Na_2_S), and 24.95 μmolh^−1^g^−1^ (0.1 M Na_2_S). The ZnIn_2_S_4_ nanosheets with appropriate Na_2_S concentrations can exhibit the highest HER under visible-light irradiation.

The optimal pH level for photocatalytic hydrogen production is primarily determined by the characteristics of the sacrificial agent and its affinity for adsorption onto the surface of the photocatalyst [49]. The impact of pH values on the photocatalytic efficiency of ZnIn_2_S_4_ nanosheets is illustrated in Figure 5d. The average HER values of ZnIn_2_S_4_ nanosheets were recorded as follows: 134.5 (pH = 1), 143.9 (pH = 3), 54.4 (pH = 6), 31.25 (pH = 9), 29.55 (pH = 12), and 16.3 μmolh^−1^g^−1^ (pH = 12.9, without adjustment). An increase in the pH value from 1 to 3 significantly enhanced the photocatalytic hydrogen production efficiency. This enhancement can be attributed to the increased dissociation of HS^−^ and S^2−^ species with the increasing pH value [47]. However, low pH values may result in photocorrosion of the catalyst and a reduction in hydrogen production efficiency [35]. Hence, the ZnIn_2_S_4_ nanosheets exhibited the highest photocatalytic hydrogen production efficiency at pH = 3.

**Figure 6:**
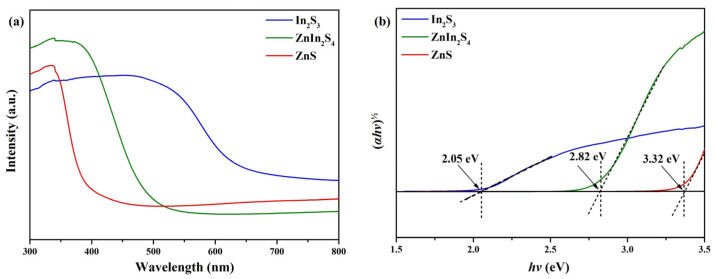
(**a**) UV–vis diffuse reflectance spectra and (**b**) Tauc plot of In_2_S_3_, ZnIn_2_S_4_, and ZnS nanosheets.

The UV–vis diffuse reflectance spectra of In_2_S_3_, ZnIn_2_S_4_, and ZnS nanosheets within the range of 300–800 nm are illustrated in Figure 6a. For In_2_S_3_ nanosheets, a pronounced absorption can be observed below 600 nm, indicating strong intrinsic interband transition absorption specific to In_2_S_3_. For ZnIn_2_S_4_ nanosheets, a notable absorption can be observed below 500 nm, corresponding to the intrinsic interband transition absorption of In_2_S_3_. Finally, for ZnS nanosheets, a significant absorption can be observed below 400 nm in the UV region, attributed to the intrinsic interband transition absorption of ZnS. These observations indicate that both In_2_S_3_ nanosheets and ZnIn_2_S_4_ nanosheets exhibit substantial absorption of visible light. The direct optical bandgap, E_g_, of the materials was determined by employing the equation (αhν)^2^ = A(hν − E_g_), where hν represents the photon energy in electron volts (eV), α is the absorption coefficient, and A is a material constant [50]. This analysis was conducted based on the data presented in Figure 6b. Consistent with prior findings, the results indicate that the In_2_S_3_, ZnIn_2_S_4_, and ZnS bandgaps were measured to be 2.05 eV, 2.82 eV, and 3.32 eV, respectively [25,51,52].

**Figure 7:**
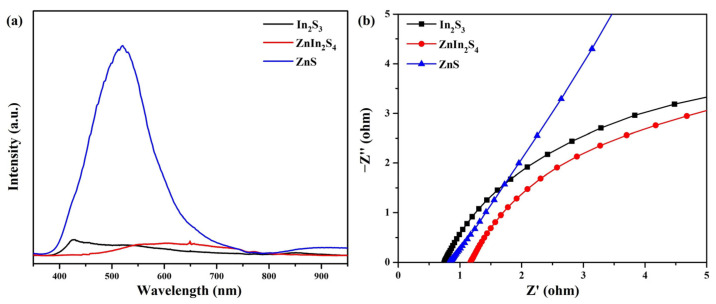
(**a**) PL and (**b**) EIS spectra of In_2_S_3_, ZnIn_2_S_4_, and ZnS nanosheets.

Photoluminescence (PL) spectra are a valuable tool for examining photogenerated charge carriers’ trapping, migration, and transfer efficiency in semiconductors [53,54]. By analyzing the PL spectra, it is possible to gain insights into the recombination and annihilation processes of photogenerated electron–hole pairs in semiconductors [55]. Figure 7a displays the PL spectra of In_2_S_3_, ZnIn_2_S_4_, and ZnS nanosheets. The ZnS nanosheets demonstrate more pronounced emission characteristics than the In_2_S_3_ and ZnIn_2_S_4_ nanosheets, suggesting a higher degree of recombination of photogenerated charge carriers within the photocatalyst. Electrochemical impedance spectroscopy (EIS) is presented in Figure 7b. The arc radii of the EIS Nyquist curves of the samples were in the following order: ZnS > In_2_S_3_ > ZnIn_2_S_4_. ZnIn_2_S_4_′s arc radius was smaller than those of In_2_S_3_ and ZnS. The smaller radius observed in the EIS test indicates lower charge transfer resistance and higher efficiency in separating charge carriers, suggesting a faster electron transfer process [56,57,58]. Consequently, the EIS results confirm that ZnIn_2_S_4_ nanosheets with sulfur vacancies facilitate the migration of photogenerated charge carriers, resulting in enhanced photocatalytic activity for hydrogen evolution.

**Figure 8:**
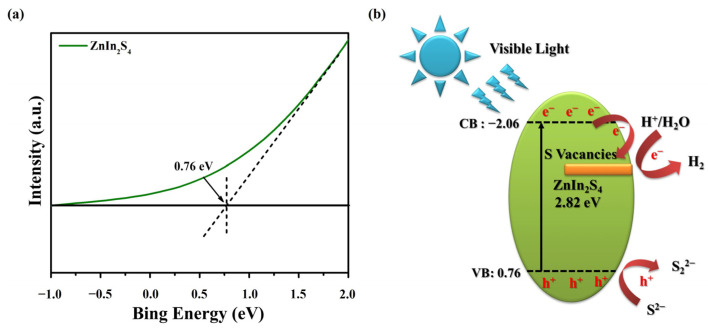
(**a**) VB XPS spectrum of the ZnIn_2_S_4_ nanosheets. (**b**) The schematic diagram depicts the charge separation process and photocatalytic reaction in the ZnIn_2_S_4_ nanosheets under visible-light irradiation.

The band structure greatly influences the generation and migration of charge carriers. Therefore, the valence band (VB) XPS was conducted on the obtained ZnIn_2_S_4_ nanosheets to investigate the valence band location. Figure 8a illustrates the typical valence band density of states (DOS) characteristics of ZnIn_2_S_4_ nanosheets with the valence band position at 0.76 eV. Based on the equation E_CB_ = E_VB_ − E_g_ and the measured bandgap of ZnIn_2_S_4_ (Figure 6b), the conduction band (CB) potential is estimated to occur at −2.06 eV. After carefully examining the findings above, we developed a hypothetical reaction mechanism for photocatalytic hydrogen production using ZnIn_2_S_4_ nanosheets. The proposed mechanism is visually depicted in Figure 8b, providing a realistic representation of the process. The presence of sulfur vacancies in ZnIn_2_S_4_ leads to the formation of defect levels within the conduction band (CB). In addition, these sulfur vacancies act as electron traps, effectively capturing photogenerated electrons and preventing their direct recombination with photoinduced holes. The trapped electrons in the sulfur vacancies of ZnIn_2_S_4_ can subsequently participate in the reduction of 2H^+^ to produce H_2_. At the same time, the photogenerated holes in ZnIn_2_S_4_ can oxidize sulfide ions (S^2−^, sodium sulfide dissociated).

**Figure 9:**
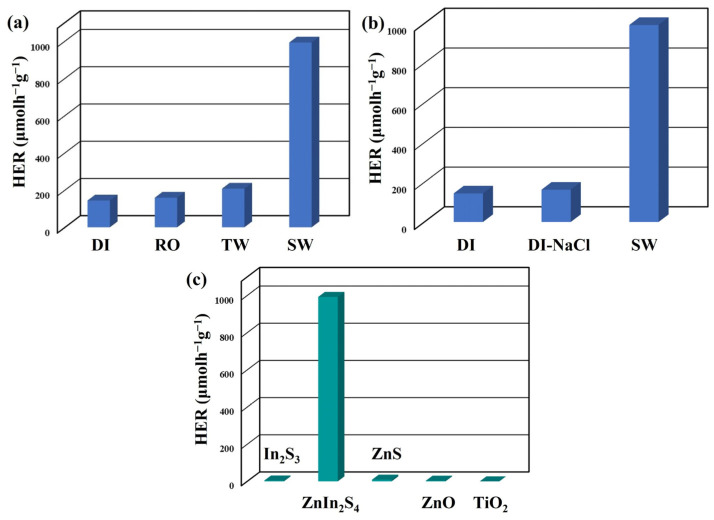
(**a**) The average HER of ZnIn_2_S_4_ nanosheets for the different water sources. (**b**) The average HER of ZnIn_2_S_4_ nanosheets for DI, DI-NaCl, and seawater. (**c**) The average HER of different photocatalysts for seawater.

Given that approximately 93% of the Earth’s water exists in the form of seawater, utilizing seawater as a source for hydrogen production through water splitting offers a practical solution to conserve freshwater resources for various purposes, such as agriculture, industry, and human consumption [39,59]. Furthermore, the hydrogen produced through the photocatalytic decomposition of seawater can be utilized to generate pure water through a fuel cell generator, adding to its potential benefits. The exceptional versatility of ZnIn_2_S_4_ nanosheets is demonstrated by their photocatalytic capability to generate hydrogen using various water sources. As depicted in Figure 9a, the dispersion of ZnIn_2_S_4_ nanosheets in 50 mL of different water sources, including deionized water (DI), reverse-osmosis water (RO), tap water (TW), and seawater (SW), was observed under visible-light irradiation with a pH value of 3 and the presence of 0.1 M Na_2_S as a scavenger. The average HER values of ZnIn_2_S_4_ nanosheets were measured as 143.9 (DI), 158.9 (RO), 207.7 (TW), and 992.4 µmolh^−1^g^−1^ (SW). With the increasing complexity of the water matrix, there is a gradual enhancement in the efficiency of photocatalytic hydrogen production. In order to investigate the influence of sodium chloride (NaCl) on the photocatalytic effect, this study included the addition of 35 psu NaCl (35 g/L) to deionized water to assess its impact on hydrogen production, as shown in Figure 9b. This result indicates that adding sodium chloride to deionized water did not significantly improve the efficiency of photocatalytic hydrogen production. Therefore, it can be inferred that sodium chloride might not be the primary factor for enhancing photocatalytic hydrogen production. The average HER values of photocatalysts, such as In_2_S_3_ nanosheets, ZnIn_2_S_4_ nanosheets, ZnS nanosheets, ZnO nanopowder, and TiO_2_ nanopowder, were assessed under visible-light irradiation using 0.1 M Na_2_S as a sacrificial reagent in seawater with a pH value of 3, as depicted in Figure 9c. The average HER values of the photocatalysts were measured as 4.74 (In_2_S_3_ nanosheets), 992.4 (ZnIn_2_S_4_ nanosheets), 9.3 (ZnS nanosheets), 3.7 (ZnO nanopowder), and 0 µmolh^−1^g^−1^ (TiO_2_ nanopowder). The ZnIn_2_S_4_ nanosheets exhibited the highest photocatalytic hydrogen production from seawater, which was about 209.4 and 106.7 times higher than that of In_2_S_3_ nanosheets and ZnS nanosheets, respectively. These results demonstrate that the microwave-assisted synthesis method promotes the formation of ZnIn_2_S_4_ nanosheets with sulfur vacancies, significantly enhancing their photocatalytic activity for hydrogen production from seawater.

**Figure 10:**
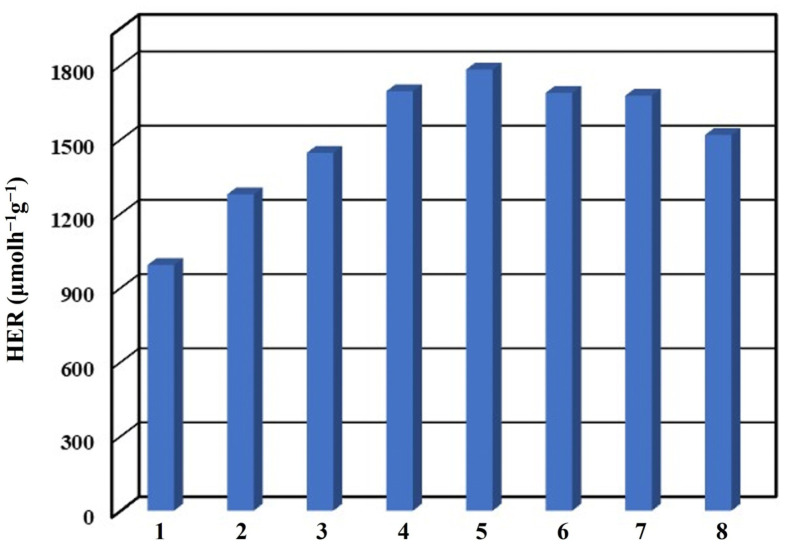
Reusability test of ZnIn_2_S_4_ nanosheets for eight cycles under visible-light irradiation.

**Figure 11:**
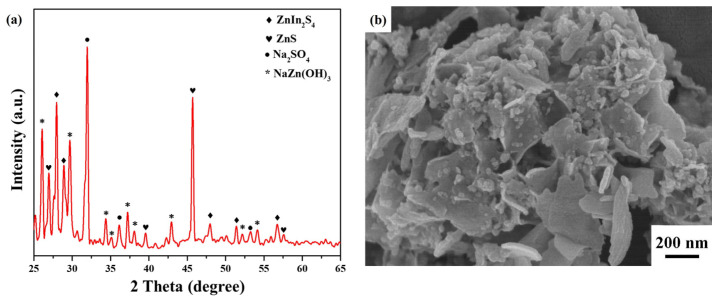
(**a**) XRD pattern and (**b**) FESEM image of ZnIn_2_S_4_ nanosheets after the eighth cycle.

The reusability of ZnIn_2_S_4_ nanosheets was investigated through an eight-cycle photocatalytic process, wherein the seawater containing 0.1 M Na_2_S at pH = 3 was renewed and subjected to visible-light irradiation, as shown in Figure 10. As a result, the average HER values of ZnIn_2_S_4_ nanosheets were recorded as follows: 992.4, 1277.5, 1445.6, 1694.8, 1782.3, 1688.2, 1676.9, and 1518.0 μmolh^−1^g^−1^. The photocatalytic hydrogen production efficiency from seawater exhibited an initial increase followed by a gradual decline as the number of cycles increased. The HER of ZnIn_2_S_4_ nanosheets showed a notable increase of 1.8 times after the fifth cycle compared to the first cycle. A noticeable distinction was observed between the XRD spectrum before the cycle (Figure 1b) and after the eighth cycle (Figure 11a). Notably, three additional crystal phases of ZnS (JCPDS card No. 72-0163), Zn_2_SO_4_ (JCPDS card No. 86-0802), and NaZn(OH)_3_ (JCPDS card No. 87-0762) were identified following the cycling process. This phenomenon can be attributed to the interaction between ZnIn_2_S_4_ nanosheets and seawater, forming ZnIn_2_S_4_/ZnS nanocomposites. ZnS possesses sulfur vacancies at the defect level, which can facilitate the migration of photogenerated charge carriers to enhance photocatalytic performance [60,61]. The morphology of ZnIn_2_S_4_ nanosheets exhibited similarity before the cycle (Figure 2b) and after the eighth cycle (Figure 11b), and noticeable changes were obtained in the roughness of the surface following the cycling process. However, a slight decrease in HER was observed in the sixth cycle, possibly due to the loss of photocatalysts during multiple centrifugations. Furthermore, these results confirm the outstanding stability and reusability of ZnIn_2_S_4_ nanosheets, highlighting their potential for wide-ranging and diverse applications in various fields.

## 4. Conclusions

This study employed a convenient and rapid microwave-assisted synthesis method to produce In_2_S_3_ nanosheets, ZnS nanosheets, and ZnIn_2_S_4_ nanosheets with sulfur vacancies. The ZnIn_2_S_4_ nanosheets demonstrated significantly enhanced photocatalytic hydrogen production performance compared to In_2_S_3_ nanosheets and ZnS nanosheets, achieving approximately 33.3 times and 16.6 times higher activity under visible-light irradiation, respectively. We further investigated the impacts of various factors, such as materials, sacrificial reagents, and pH values, on the photocatalytic hydrogen production of ZnIn_2_S_4_ nanosheets. The ZnIn_2_S_4_ nanosheets exhibited the highest photocatalytic hydrogen production efficiency from seawater, surpassing In_2_S_3_ nanosheets and ZnS nanosheets by approximately 209.4 times and 106.7 times, respectively. The presence of sulfur vacancies in ZnIn_2_S_4_ nanosheets holds great promise for developing highly efficient and stable photocatalysts for hydrogen production from seawater under visible-light irradiation. This research can potentially conserve precious freshwater resources and use light energy to split seawater into hydrogen energy.

## Data Availability

Not applicable.
